# Strategy and rationale for urine collection protocols employed in the NEPTUNE study

**DOI:** 10.1186/s12882-015-0185-3

**Published:** 2015-11-17

**Authors:** Marie C. Hogan, John C. Lieske, Chrysta C. Lienczewski, Lisa L. Nesbitt, Larysa T. Wickman, Christina M. Heyer, Peter C. Harris, Christopher J. Ward, Jamie L. Sundsbak, Luca Manganelli, Wenjun Ju, Jeffrey B. Kopp, Peter J. Nelson, Sharon G. Adler, Heather N. Reich, Lawrence B. Holzmann, Matthias Kretzler, Markus Bitzer

**Affiliations:** Department of Medicine, Division of Nephrology and Hypertension, Mayo Clinic, 200 First Street SW, Rochester, MN 55905 USA; Department of Internal Medicine – Nephrology, University of Michigan Health System, Ann Arbor, MI USA; Cardiovascular Research, Mayo Clinic, Rochester, MN USA; Pediatric Nephrology, University of Michigan, Ann Arbor, MI USA; Nephrology and Hypertension Research, Mayo Clinic, Rochester, MN USA; Department of Internal Medicine, Division of Nephrology and Hypertension, University of Kansas Medical Center, Kansas City, KS USA; Oncology, Novartis Pharma AG, Basel, Switzerland; Kidney Diseases Branch, National Institute of Diabetes and Digestive and Kidney Diseases, National Institutes of Health, Bethesda, MD USA; Division of Nephrology and Kidney Research Institute, University of Washington, Seattle, WA USA; Division of Nephrology and Hypertension, Harbor-UCLA Medical Center, Torrance, CA USA; Department of Medicine, University Health Network and University of Toronto, Toronto, ON Canada; Renal-Electrolyte and Hypertension Division, Perelman School of Medicine, University of Pennsylvania, Philadelphia, PA USA

**Keywords:** Exosome, Urinalysis, Urine specimen collection

## Abstract

**Background:**

Glomerular diseases are potentially fatal, requiring aggressive interventions and close monitoring. Urine is a readily-accessible body fluid enriched in molecular signatures from the kidney and therefore particularly suited for routine clinical analysis as well as development of non-invasive biomarkers for glomerular diseases.

**Methods:**

The **Nep**hrotic Syndrome S**tu**dy **Ne**twork (NEPTUNE; ClinicalTrials.gov Identifier NCT01209000) is a North American multicenter collaborative consortium established to develop a translational research infrastructure for nephrotic syndrome. This includes standardized urine collections across all participating centers for the purpose of discovering non-invasive biomarkers for patients with nephrotic syndrome due to minimal change disease, focal segmental glomerulosclerosis, and membranous nephropathy. Here we describe the organization and methods of urine procurement and banking procedures in NEPTUNE.

**Results:**

We discuss the rationale for urine collection and storage conditions, and demonstrate the performance of three experimental analytes (neutrophil gelatinase-associated lipocalin [NGAL], retinol binding globulin, and alpha-1 microglobulin) under these conditions with and without urine preservatives (thymol, toluene, and boric acid). We also demonstrate the quality of RNA and protein collected from the urine cellular pellet and exosomes.

**Conclusions:**

The urine collection protocol in NEPTUNE allows robust detection of a wide range of proteins and RNAs from urine supernatant and pellets collected longitudinally from each patient over 5 years. Combined with the detailed clinical and histopathologic data, this provides a unique resource for exploration and validation of new or accepted markers of glomerular diseases.

**Trial registration:**

ClinicalTrials.gov Identifier NCT01209000

**Electronic supplementary material:**

The online version of this article (doi:10.1186/s12882-015-0185-3) contains supplementary material, which is available to authorized users.

## Background

There is currently considerable interest in developing urine procurement protocols for longitudinal clinical studies that collect and process large numbers of samples from patients with kidney disease, and specifically with various glomerular diseases. Utilizing these samples for diverse research questions in both basic and clinical studies of glomerular disease could help to advance the understanding of glomerular disease pathogenesis [1]. However, few, if any, published reports discuss the rationale or strategy for serial urine collections and optimal storage conditions [2–6]. Those published studies that do so focus on subcomponents of urine, and examine only limited numbers of abundant analytes. Furthermore, little rationale or strategy exists in these prior studies to inform the protocols needed for multicenter clinical studies [2].

NEPTUNE (Clinical trials.gov: NCT01209000) is a multicenter study that is identifying and prospectively following 450 incident, biopsy-proven cases of nephrotic syndrome (NS) from membranous nephropathy (MN), minimal change disease (MCD), or focal segmental glomerulosclerosis (FSGS) (or other glomerulopathies groups/OG if none of the above). Currently, 21 academic clinical centers in the United States and Canada are participating in the NEPTUNE protocol [7] designed for systems biology approaches to phenotype-genotype correlations. As part of the NEPTUNE protocol, urine samples are being collected at 11 time points over 30 months for clinical outcome measures (total protein, albumin excretion), as well as to establish a urine Biobank for further ancillary studies on these rare diseases.

Urine is a logical place to identify potential biomarkers of kidney disease. Urinary neutrophil gelatinase-associated lipocalin (NGAL, *LCN2*) is up-regulated after tubular cell injury and may also be a marker of kidney disease and severity in chronic kidney disease, including FSGS [8, 9]. Levels of NGAL may differentiate the steroid sensitive from the steroid resistant form and correlate with disease severity in steroid resistant nephrotic syndrome [10]. Alpha-1 microglobulin (AMBP; *AMBP*) and retinol binding protein (RBP) are low molecular weight markers of proximal tubular dysfunction [11–13]. Alpha-1 microglobulin has been proposed to correlate with the extent of tubulointerstitial damage in membranous nephropathy and to have a predictive value for functional outcome and response to therapy that is superior to 24-h proteinuria [14]. Urinary RBP is also a marker of tubulointerstitial disease [12, 15]. Podocalyxin (PODXL), fibrocystin (FIBRO), polycystin 1 (PC1) and smoothened (SMO) are implicated in the glomerular or tubular disorders and present in urine or its exosome subfraction in low abundance but detectable with well-validated antibody reagents that were available to us at the time these studies were performed [16–22]. RNA obtained from urine cellular pellets, microvesicles, and urine metabolites are now showing promise for identification of disease biomarkers [23–25].

Here, we discuss some of the challenges in implementing a urine collection protocol across multiple clinical sites. We present results that define storage and preservation conditions that would allow for analyses of urine proteins (especially proteomic studies), RNA, and DNA, from both soluble urine fractions and exosomes, as sources of biomarkers of interest to the nephrology community [6, 26, 27].

## Methods

### Urine stability studies

Stability studies were performed on waste urine obtained from the Mayo Renal Function Laboratory. Urines (25–50 mL) were retrieved within 2 h of collection from patients with and without proteinuria without preservative (random samples). Contaminated samples, assessed as a positive Gram’s stain, were not used. Samples were studied over 1–7 days under the following preservative conditions: 1) ambient (room temperature [RT] without preservative), 4 °C, and −20 °C without preservative; 2) ambient (RT) with addition of toluene, thymol, acetic acid, boric acid, 6 N HCl 50 % acetic acid; 3) addition of Roche Complete (no EDTA) and Sigma protease inhibitors and samples either immediately then frozen at −80 °C or exosomes extracted and stored in 100 μL of 0.25 M Sucrose 20 mM HEPES (pH 7.4) with added Complete at −80 °C. Up to 10 independent urine samples were assessed for each biomarker studied. Biomarkers were measured at time zero, 1, 3 and 7 days after urine collection. Biomarkers were considered stable if mean values were within 20 % of time zero measurements. The Mayo Clinic Institutional Review Board approved studies using waste urines; therefore, informed consent was waived (IRB # 09–004285). All NEPTUNE (Clinical trials.gov: NCT01209000; date of entry July 29, 2010) local sites obtained IRB approval and patients provided informed consent or parental assent for urine collections; located at University of Michigan Medical Center, New York University Langone Medical Center, Johns Hopkins Medical Institute, John H. Stroger Cook County Hospital, University Health Network – Toronto, University Hospitals Case Medical Center, University of Southern California Lost Angeles Children’s Hospital, Los Angeles Biomedical Research Institute, Steven and Alexandra Cohen Children’s Medical Center of New York, The Mayo Clinic, Children’s Hospital at Montefiore Medical Center, University of Miami - Miller School of Medicine, University of North Carolina at Chapel Hill, Children’s Hospital of Philadelphia, Seattle Children’s Hospital, Providence Medical Center, NIDDK, Columbia University, Temple University, Emory University - Children’s Hospital of Atlanta, Stanford University, University of Texas at Southwestern.

### Candidate biomarkers

#### Soluble proteins

NGAL was measured using a Bioporto Rapid ELISA [28]. AMBP and RBP were measured on a Siemens BN II nephelometer using kits from Siemens and The Binding Site, respectively.

#### Cellular pellet proteins

**Fig. 1:**
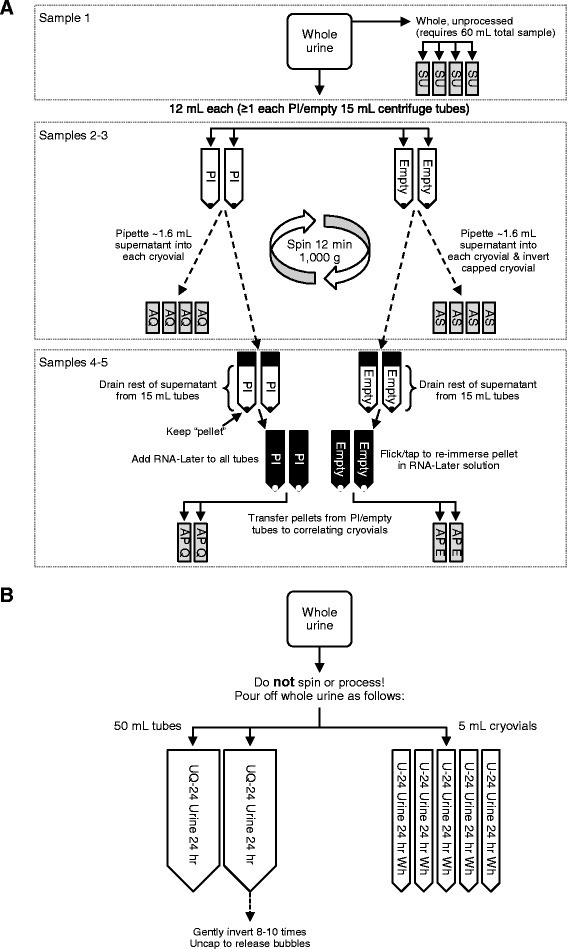
**a** NEPTUNE spot urine sample processing: Sample 1: 4 × 2 mL cryovials whole unprocessed “raw” urine (SU). Sample 2: 4 × 2 mL samples with sodium azide additive (AS). Supernatant after low speed centrifugation is saved in cryovials with 20 μL 100 mM sodium azide (12 mL × 4). Sodium azide has anti-bacterial properties. Sample 3: 4 cryovials with proteinase inhibitor (PI). Sample 4: 2 sets of pellet from this are stored in RNA*later* obtained by centrifugation at 1000 g × 12 min in a tabletop centrifuge (AP-E). Sample 5: 4 × 2 mL from the PI sample. 2 sets of pellet from this are stored in in RNA*later* obtained by centrifugation at 1000 g x 12 min in a tabletop centrifuge (AQ-Q). All samples are stored on ice in transport and during collection. All samples are frozen as soon possible at −80 °C. Cryovial final volume stored is 1.6 mL urine. **b** NEPTUNE 24-h urine sample processing. One subaliquot 40 mL whole urine (inverted x 3) frozen at −80 °C immediately (U1). 1 subaliquot (40 mL) of whole urine with protease inhibitor (6 μL; Sigma P1860) (UQ1); 5 × 5 mL cryovials whole urine (U-24)

PODXL, FIBRO, and SMO were examined in urine pellets across several storage conditions with and without preservatives collected using the NEPTUNE protocol (Fig. 1, Additional Methods-Manuals of Procedures for Spot Urine and for 24-h Urine Processing).

#### Cellular pellet DNA

To examine DNA quality from cellular pellets, the large *PKD1* gene in a region of the genome that is highly repetitive was examined using the robust Multiplex Ligation-dependent Probe Amplification (MLPA) method which is a multiplex PCR method detecting abnormal copy numbers of genomic DNA; commercial kits are available for up to 50 genes. In this commercially-available kit, oligonucleotide probes are designed to make the ligated base overlie the site of sequence differences among highly similar sequences allowing discrimination of unique sequence copy numbers, hence giving us a way to determine DNA quality across the genomic region of *PKD1* for the first time in urine cellular pellets and permitting study of somatic mutations in candidate genes in the NEPTUNE cohort.

#### Cellular pellet RNA

To examine urine RNA expression, the frozen urine pellet was thawed on ice, and either RNA was isolated directly, or pellet was washed by suspending it in 1.5 mL DEPC-treated PBS, transferred to a 1.6 mL Eppendorf tube, and then centrifuged at 13,000 g for 5 min at 4 °C. We examined pellet RNA isolated from urine cellular pellets, and RNA was isolated using the column-based method (i.e., RNeasy, Qiagen) at Mayo Clinic; the NEPTUNE pellets were studied in Michigan. For detection of mRNAs, qRT-PCR was performed for podocin, nephrin, aquaporin2, and TGF-β1 as described [23]. MicroRNAs were detected using TaqMan® microRNA arrays (Applied Biosystems) as described [29].

## Results

### NEPTUNE urine collection protocol

A defined urine collection procedure (Table 1; Additional file 1: Table S1; and Additional Methods-Manuals of Procedures for Spot Urine and for 24-h Urine Processing) was created based upon best practices and literature review [30, 31]. This approach was employed to provide a standardized collection procedure applied to all participating centers. Samples are collected using two working protocols: (1) from 24-h whole urine collection, and (2) spot urine collections (recorded as “am” or “pm” void). A total of 13 visits are planned for the anticipated 600 participants in the study. In each visit, a total of 11 tubes will be generated: three 5 mL tubes (*n* = 4 times), seven 2 mL tubes (*n* = 12), and one 50 mL tube (*n* = 2). In other words, a total of 121 samples will be generated per participant over 13 visits. To date, about 400 participants have been enrolled in the study. We anticipate there will be up to 72,600 urine samples collected through the study timeframe.

**Table 1:** NEPTUNE Urine Collection Timeline

Visit Number^a^	V1	V2	V3	V4	V5	V6	V7	V8	V9	V10	V11	V12	V13
Eligibility assessment	X												
Baseline H&P		X											
Follow-up H&P				X	X	X	X	X	X	X	X	X	X
Biosample baseline		X											
Biosample follow-up				X	X	X	X	X	X	X	X	X	X
24-hour urine		X		X	X	X		X		X		X	X
Clean catch/(Spot) urine		X		X	X	X	X	X	X	X	X	X	X
Renal biopsy tissue			X^b^										

Abbreviations: *H&P* history and physical examination, *UA* urinalysis, macroscopic (color, appearance, specific gravity, pH, leukocyte esterase, nitrite, protein, glucose, ketones, urobilin, bilirubin, blood), *V* visit

^a^There will be a total of 13 visits for the anticipated 600 participants in the study. In each visit, a total of 11 tubes will be generated (3 of 5 mL tubes, 7 of 2 mL tubes and 1 of 50 mL tube)

^b^Renal biopsy includes a pre-biopsy 10 mL spot urine

### Study dependent urine based outcomes

To determine the rates of two clinical outcomes, change in urinary protein excretion and change in kidney function will be determined in both major disease groups (MCD/FSGS and MN that are fully histopathologically characterized; Additional file 1: Table S2). The urine protein measures will be performed at a single Central Reference Laboratory (Mayo Clinic, Rochester, Minnesota). A 24-h urine protein quantitation will be performed at each clinical site per this protocol for real time clinical care. The urine outcome will be performed on one of the three 5 mL tubes for each collection. A spot and 24-h urine albumin, creatinine (gold standard) and total protein will be measured by the Central Reference lab for the study primary outcomes. Spot urines for protein/creatinine ratio will also be obtained according to the study protocol and compared with the values from the 24-h urines. Each enrollee receives a unique patient identifier (PID), which is applied to all components of their collected urine samples (Additional file 1: Table S2). The NEPTUNE study, including the urine collection protocol, was approved by the Institutional Review Boards at all participating institutions (currently at 21). The study was conducted in adherence to the Declaration of Helsinki. Written informed consent for participation in the study was obtained from participants or, where participants are children, a parent or guardian.

### Local center collections

Clinical Research Units are used at each site for these standardized biosample collections. All sites have received standardized training. Special attention will be given to facilitate the collection of urines in pediatric patients enrolled in this study. For incontinent infants and children, urine may be collected using a urine collection bag for a spot sample or a timed void.

### Urine protein stability

#### Soluble protein biomarkers

**Fig. 2:**
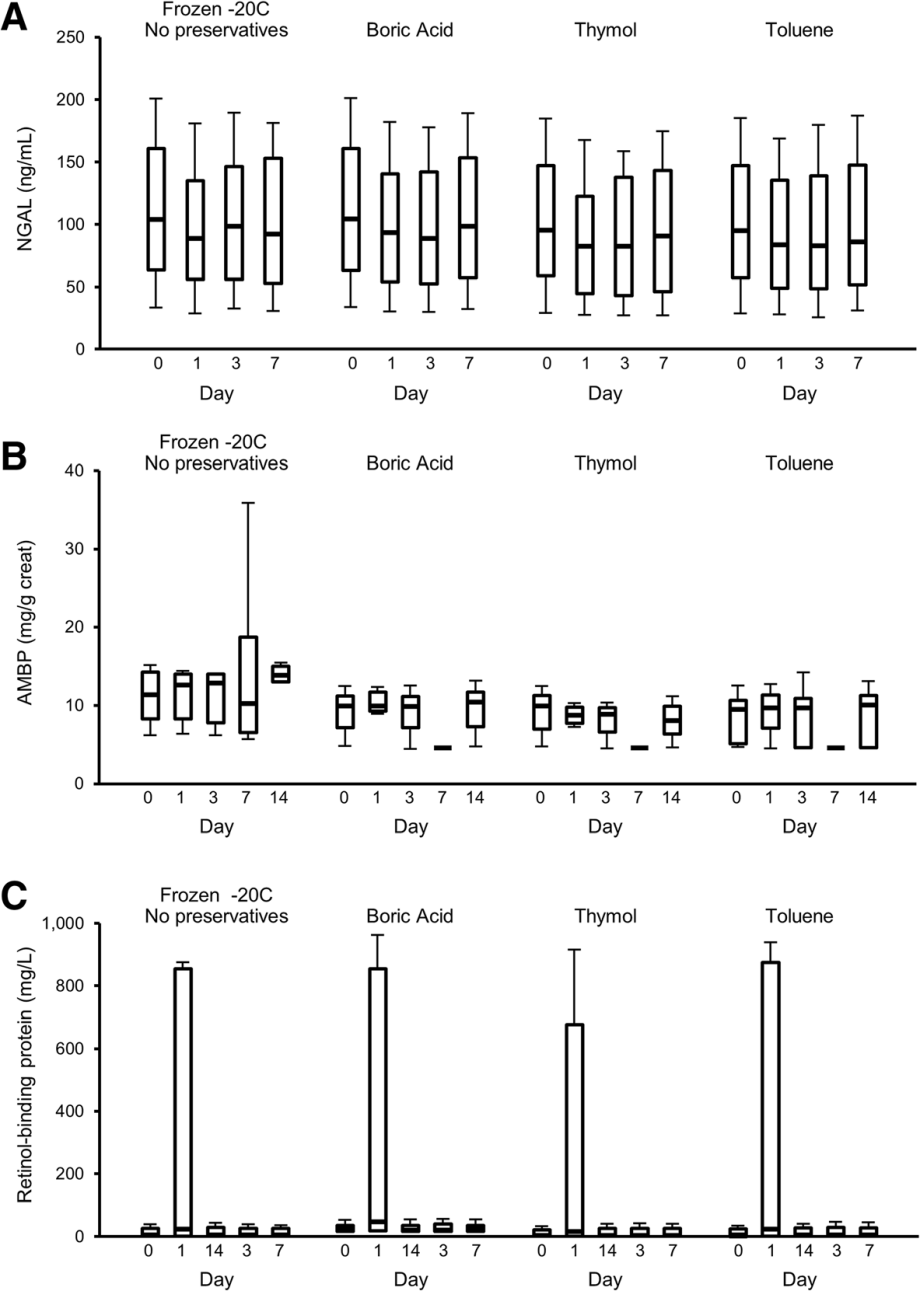
Immunoassay of soluble proteins. **a** Neutrophil gelatinase-associated lipocalin (NGAL) (**b**). Alpha-1 microglobulin (AMBP). **c** Retinol-binding globulin. All 3 were stable at room temperature, 4 °C, and −20 °C in urine (shown in this figure) without preservatives and also with toluene, thymol, and boric acid. None were stable with acetic acid, 6 N HCl, or 6 N HNO_3_ (Additional file 2: Table S3). Median and interquartile ranges are shown

All 3 proteins tested (NGAL, AMBP, RBP) were stable for up to 7 days at RT, 4 °C, and −20 °C (Fig. 2a-c). They were also stable with toluene, thymol, and boric acid preservatives, but not with acetic acid, 6 N HCl, or 6 N HNO_3_ (Additional file 2: Table S3). These 3 proteins were stable for up to 3 freeze/thaw cycles at −20 °C. Therefore, urine samples stored frozen without preservative will be adequate for selected urine proteins [32]. Other biomarkers susceptible to urinary proteases may require more specialized handling (see below).

#### Cost analysis and protein stability studies with protease inhibitors

To collect suitable samples for future detailed proteomics studies, we evaluated two commercially-available protease inhibitors: Sigma protease inhibitor P 1860 TM (Sigma-Aldrich Corp., St. Louis, MO) and Roche Complete mini protease inhibitor (Complete, EDTA free, 11873580001, Roche Diagnostics Corp, Indianapolis, IN) and examined several proteins of interest by Western analysis (Additional file 1: Figure S1). We found the Sigma protease cocktail was not only more efficient in preserving the proteins of interest, but that it was also significantly less expensive, as it could be divided for use across the sub-aliquots being collected for the future proteomics studies of this trial (Additional file 1: Table S1). Indeed 20 % less Sigma protease inhibitor was just as efficient as the recommended amounts by the manufacturer in preserving the proteins that we tested (Additional file 1: Figure S1). The protocol employs 6 μL per 50 mL urine contained (containing 40 mL urine = 4.8 μL/40 mL).

#### Cellular pellet proteins

We also examined protein stability in cellular pellets for selected proteins using Western analysis, with and without the above protease inhibitors (Additional file 1: Figure S1). There was substantially better detection of the podocyte marker of interest PODXL in cellular pellets stored with protease inhibitors, and again found that Sigma protease inhibitor product may in fact be superior to the more expensive Roche protease inhibitor cocktail (Additional file 1: Figure S1). Without the presence of protease inhibitor, the proteins we examined (podocalyxin, fibrocystin and smoothened) did not survive 12 months of storage (6 month representative Western shown), or a freeze/thaw cycle at −80 °C. Fibrocystin and smoothened were only variably preserved under any conditions tested.

#### Exosomal proteins

**Fig. 3:**
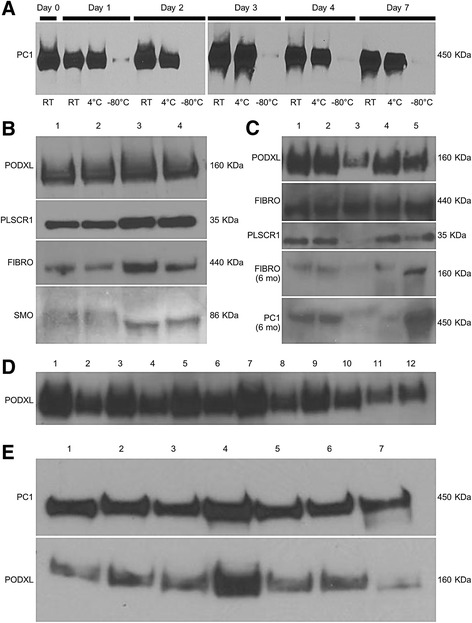
Western blot of exosome pellets. Exosomal proteins of interest including podocalyxin (PODXL), fibrocystin (FIBRO) and polycystin 1 (PC1) were examined by Western blot after storage and compared with freshly-isolated exosomes. These proteins were best preserved by the Sigma protease cocktail. **a** Polycystin 1 detection by Western blot was stable for 1 week at room temperature after exosomes were isolated and stored in 0.01 % sodium azide (*n* = 7). However, polycystin 1 was not detected well from exosomes isolated after urine was frozen.at -80C probably because of precipitation issues and unlikely due to loss of protease activity. Effects of preservatives on Western blot of exosome pellet proteins PODXL, scramblase (PLSCR1), FIBRO, and smoothened (SMO). **b** Lane 1, Fresh, no preservatives; Lane 2, Fresh, no preservatives; Lane 3, −80 °C Frozen/Thawed; Lane 4, −80 °C Frozen/Thawed. **c** Comparison of varied amounts of protease inhibitors on exosome protein detection by Western blot. Initial assessment and following 6 months storage at −80 °C. Lane 1, 4.8 μL. Sigma protease inhibitor; Lane 2, 4.0 μL Sigma protease inhibitor; Lane 3, 1:100 Roche Complete tablet; Lane 4, −80 °C Frozen/Thawed; Lane 5, Fresh, no preservatives. **d** Exosomal Podocalyxin fared well at RT and at 4 °C. Sodium azide did not affect their survival. **e** Analysis of exosomes extracted from frozen (−80 °C) raw urine stored for 12 months

To evaluate the impact of freezing on the integrity of exosomes in stored urine, we compared exosome preparations after raw urine was stored at RT, 4 °C, or −80 °C for 0, 1, 3, or 7 days. The urine exosome protein PC1 was readily detected from urine stored at RT and 4 °C for up to 7 days, but there was a marked decrease in detectable protein in the urine thawed after any period of −80 °C storage. However, exosomal proteins in the samples stored at RT and 4 °C were detectable out to day seven (Fig. 3), which might permit shipping of such samples from a clinical site to a research laboratory for subsequent isolation of exosomes. The loss of the PC1 with storage at −80 °C is likely due to protein aggregation, which led to loss of the exosome fraction after thawing, and a low-speed spin done to pre-clear the urine sample in preparation for ultracentrifugation.

Several exosomal proteins were examined (PODXL, FIBRO, and PC1) by Western blot in exosomes stored with protease inhibitor in comparison to freshly isolated exosomes. We found that PC1 is stable for 1 week after isolation of exosomes when stored (in 0.01 % sodium azide with protease inhibitor tablet [Roche]) were stable at RT and 4 °C (Fig. 3a of representative data). FIBRO was well preserved in exosomes stored with or without protease inhibitor, whereas it was not well detected in cell pellets under these conditions, especially with storage. Scramblase (PLSCR1) was well preserved with Sigma protease inhibitor and one freeze/thaw cycle. As with cellular pellets, in general, addition of protease inhibitor was essential to protein survival (Fig. 3b-d, Additional file 1: Figure S1).

#### Long-term (≤twelve month storage data) for exosomes extracted from Raw urine

The exosomal proteins SMO, PODXL, FIBRO, PLSCR1 and PC1 were detected in exosomes that were isolated from fresh urine, then stored at −80 °C for 12 months, without any evidence that they degraded (Fig. 3e).

#### DNA integrity from cellular pellets

The MLPA technique allows simultaneous screening of multiple target sequences in a single reaction by using pairs of probes that carry tails for binding of common amplification primers. To test if this technique could be applied to evaluate disease gene transcripts present in urine pellets, we examined the large polycystic kidney disease gene *PKD1* transcript by MLPA and long-range PCR and found that it was readily detectable in urine pellet DNA (Additional file 1: Figure S2A). The quality of the DNA was compatible with that seen from blood and buccal smear DNA.

#### RNA integrity cellular pellet and exosomes

RNAs that reflect the population of epithelial cells lining the urinary tract can be reproducibly recovered from a centrifuged urine pellet. Cellular pellet RNA quality was not as consistent across samples collected from our clinical laboratory, or in comparison to RNA from freshly isolated chinese hamster ovary cells grown in cell culture (used as control) (Additional file 1: Figure S2B, C, D). We determined that a washing step should be included to increase recovery of RNA (manuscript in preparation, Wickman). However, RNA could still be recovered if the pellet had been frozen at −80 °C in RNA*later®* and the washing step was subsequently performed on the thawed sample, although recovery of RNA was reduced on average by about 50 %. For low level transcripts (e.g., podocin), a urine volume of at least 30 mL will provide a measurable signal in 80 % of normal urine samples. A starting urine volume of less than 30 mL of urine increases the undetectability rate. Urine mRNA amount recovered decays by about 50 % over the first 4 h following voiding, but then remains quite stable for up to 24 h at 4 °C, compatible with the concept that a proportion of voided cells remain intact within the urine milieu as a potential source of RNA [23]. Abundant mRNAs, including TGF-β1 and aquaporin 2, were detected in 95 % of samples tested, whereas less abundant mRNA transcripts for nephrin and podocin were detected in 88 % of samples. Small RNA from cellular pellets was detected (Additional file 1: Figure S2F,G). Out of 384 microRNAs tested, 38 and 169 microRNAs were detected with a CT-value less than 32 (robust detection) and 40 (borderline detection), respectively, using a qRT-PCR array. Thus, the urinary pellet is suitable for evaluation of RNA transcripts and microRNAs, but is limited by transcript abundance [23]. Alternatively, in exosome subfractions which have been reported to be enriched in small RNAs (including microRNAs), we detected robust and consistent small RNA content (Additional file 1: Figure S2E) as others have observed [33–38], and have begun their characterization using next generation sequencing.

## Discussion

The NEPTUNE study is a multi-center trial that includes the goal to establish a biorepository for future research studies, including biomarker discovery. We have developed and implemented a uniform urine collection and storage protocol for all study sites. The goal of this protocol is to obtain and store urine samples and specific subfractions suitable for biomarker research in a well-defined and large disease cohort. Stability data for several promising candidate urinary biomarker proteins (NGAL, RBP, and AMBP) demonstrate they can be reliably measured in urine without preservatives and at storage temperatures as low as −80 °C [8, 39]. Other markers or urinary subfractions (e.g., exosomes) appear to require use of protease inhibitors for long-term storage.

While we have not exhausted every possible storage scenario or its effect on every potential urine protein biomarker, we have incorporated current best practices with some consideration given to cost. Based on this and other published data, frozen urine specimens preserved at −80 °C should be adequate to study many soluble urine biomarkers, including albumin [40].

The current study also demonstrates how the protocol could be applied to the study of biologically-rich exosome-like vesicles which contain proteins of podocyte origin and more than 5,000 proteins and abundant miRNA [35, 41–44]. Currently, our collection protocol stores a subgroup of urine subfractions with protease inhibitor that are immediately frozen. This process appears suitable for soluble protein analysis, and it may permit samples to be used for urine exosome analysis [45]. Storing unprocessed raw urine or urine which has been pre-spun at 4000 g for 15 min at 4 °C is not optimal for exosome recovery, as demonstrated by the poor yield of PC1 after freezing at −80 °C (Fig. 3). This may be because of the tendency for proteins and salts to precipitate out of urine as it thaws. We hypothesize that this precipitate could interfere with purification by trapping exosomes in the initial low speed spin down. Conversely, the current studies suggest that proteins are stable for Western blot analysis in purified exosomes when stored at −80 °C for up to twelve months. Indeed, our preliminary analysis of urine stored at RT for up to 7 days indicates that even under these conditions, samples might be suitable for Western analysis of urinary vesicles.

To date, use of nanomembrane concentrators have yielded exosome proteins of “very low purity,” is laborious (~130 min), and not uniform, although it does avoid the need for ultracentrifugation, which is impractical in a clinical setting [22, 35, 46, 47]. Studies report use of these concentrators on frozen raw urine to examine a few selected exosome markers, but to date, all more extensive exosome proteomic studies have employed ultracentrifugation protocols and fresh urine [22, 42, 44, 45, 48, 49]. When a combination of nanomembrane concentrators and size exclusion chromatography was applied to nephrotic patient urine samples, highly abundant soluble proteins (e.g., albumin) obstructed the nanomembrane filter and led to identification of only a limited proteome [50]. Our study and others suggests that protease inhibitors will be required to study certain exosomal proteins [22, 44, 46, 49]. The Sigma protease inhibitor appears superior to the Roche Complete tablet, and in this study was significantly less expensive. Indeed, an even lower concentration of the Sigma protease inhibitor may be just as effective as the concentration used in our current storage protocol. Sigma protease inhibitor was also easier to use since it is a liquid and can be used on urine volumes <50 mL. A possible explanation for the lower effectiveness could include the required extra time to dissolve the tablet into solution.

There are a number of limitations to the scope of the NEPTUNE Urine collection protocol. For one, the consortium receives many requests for samples to examine the validity of exploratory urine biomarkers. Each analyte will likely require stability studies, etc., to determine optimal detection in urine, which will likely eventually exhaust our stored urine aliquots. The number of samples that could be stored at each time point for each patient had to be weighed against what was practical and financially feasible at the time the collection protocol was developed. Our solution at the time was to also store multiple aliquots from the 24-h urine collection, and these samples are also accessible to requestors.

Apart from NEPTUNE, many additional cohort studies and randomized trials are ongoing that employ protocols for urine collection and storage. To our knowledge, urine specimens collected and stored in these other protocols vary and may not provide the same utility for biomarker research as the NEPTUNE protocol. Many storage methods are sufficient for studying traditional biomarkers such as creatinine and albumin; however, it is unknown the degree to which findings generated from urine samples processed with other protocols will provide comparable results in proteomic, metabolomic, RNA, and miRNA discovery and validation studies on banked urine

## Conclusions

Currently, there is an absence of large-scale biomarker discovery and validation studies on urine collected from well-defined glomerular disease cohorts [1, 44]. Biomarkers are urgently needed to understand glomerular disease pathogenesis and to assess responsiveness versus non-responsiveness and possibly limit the duration of toxic therapies. We report here our progress in establishing a standardized, practical, and cost-effective urine procurement protocol that will facilitate research protocols on urine in the NEPTUNE cohort, permit identification of markers of diagnosis, prognosis, and responsiveness to therapy, or markers indicative of the disease. We have provided additional information on RNA and protein quality in urine subfractions. We add to current knowledge on preservation conditions in urine subfractions as they will be applied across large multicenter clinical studies. Requests are now being reviewed for use of NEPTUNE urine samples. We invite the nephrology community to submit requests for the use of these samples through the NEPTUNE Ancillary Studies process (NEPTUNE-study.org).

## Additional files

Additional file 1: Table S1. Standard Components of Two Commercially-Available Protease Inhibitors and Cost Analysis. **Tables S2.** Urine based primary outcome measures for the FSGS/MCD and MN Cohort NEPTUNE Study. **Figure S1.** Western blot of podocyte proteins in urinary cell pellets from pooled urine. **Figure S2.** (A) The MLPA and long-range PCR analysis of PKD1 DNA in urinary cell pellets. Long-range PCR of exons 13-15 (~5kb) and the nested PCR from a 1:1000 dilution of the long-range product. Nested PCR product for PKD1 Exon 15 part 5. ( ~700kb). The DNA ladder is seen in the second photograph. The loading order was Blood, Saliva, Urine, for patient R2140. Comparison analysis of RNA. Quality RNA was obtained from (B) control (RNA isolated from CHO cell line), (C & D) urine cellular pellets, (E) exosomes. RNA integrity numbers are shown from representative samples. (F) Impact on urine volume on reproducibility of mRNA yield for podocin, nephrin, aquaporin2, TGF β1 transcripts and (G) Number of samples (percent) with undetectable transcripts (patients vs. controls). Additional Methods. Additional file 2: Specimen Stability Worksheet.

## Abbreviations

AMBP: Alpha-1 microglobulin
FIBRO: Fibrocystin
FSGS: Focal segmental glomerulosclerosis
MCD: Minimal change disease
MLPA: Multiplex Ligation-dependent Probe Amplification
MN: Membranous nephropathy
NEPTUNE: The **Nep**hrotic Syndrome S**tu**dy **Ne**twork
NGAL: Neutrophil gelatinase-associated lipocalin
NS: Nephrotic syndrome
PLSCR1: Scramblase
PODXL: Podocalyxin
PC1: Polycystin 1
RBP: Retinol binding protein
RT: Room temperature
SMO: Smoothened
