# Supplementary material for: Strategy and rationale for urine collection protocols employed in the NEPTUNE study
Source: BMC Nephrol. 2015 Nov 17;16:190. doi: 10.1186/s12882-015-0185-3 (PMC4650313; doi:10.1186/s12882-015-0185-3)

Additional Table S1. Standard Components of Two Commercially-Available Protease Inhibitors and Cost Analysis.

**A. Complete ULTRA Mini Protease Inhibitor Tablets®***		
**Protease mixture**	**Enzyme concentration (mg/mL)**	**% inhibition after 60 min**
Pancreas-extract	0.027	94
Trypsin	0.0036	95
Papain	0.0030	96
Proteinase K	0.0008	95
*$187.00/30 tablets. 1 tablet/100 mL urine. Spot sample: does not work well since tablet cannot easily be divided into small portions. 24-hour sample: $6.23/patient sample.		

**B. Protease Inhibitor Cocktail (Sigma P1860)**
**Aprotinin (Product Code A 1153**)<br>Inhibits serine proteases, such as trypsin, chymotrypsin, plasmin, trypsinogen, urokinase, and kallikrein; inhibits human leukocyte elastase, but not pancreatic elastase.
**Bestatin (Product Code B 8385)**<br>Inhibits aminopeptidases such as leucine aminopeptidase and alanyl aminopeptidase.1,2,3,4E-64[trans-epoxysuccinyl-L-leucylamido(4-guanidino) butane] (Product Code E 3132)<br>Inhibits cysteine proteases such as calpain, papain, cathespsin B, cathepsin L.
**Leupeptin (Product Code L 2884)**<br>Inhibits both serine and cysteine proteases, such as calpain, trypsin, papain, and cathepsin B.
**Pepstatin A (Product Code P 4265)**<br>Inhibits acid proteases such as pepsin (human or porcine), renin, cathespin D, chymosin (bovine rennin), protease B (Aspergillus niger)
Cost: $90.10/1mL. Spot urine sample: $2.70/patient sample. 24-hour sample; $1.08/patient sample.<br>*Exact components proprietary. 05 892 953 001 (Roche product insert data)

Additional Table S2. Urine based primary outcome measures for the FSGS/MCD and MN Cohort NEPTUNE Study.

Proteinuria (U:C)After Observation period	Standard Definition	Definitions for the Cohorts
U:C ≤0.3 g	Complete remission	Complete/partial Remission
Reduction in U:C of >50% plus final P:C ≤3.5 g but >0.3 g	Partial remission	
Reduction in U:C of >50% with final P:C >3.5 g	Limited response	No response
Reduction in U:C of <50% (include increase in U:C <50%)	Non-Response	
Proteinuria increases by >50%	Progressive Proteinuria	
New development of nephrotic range proteinuria, i.e., >3.5 P:C after reaching a complete or partial remission	Relapse	Relapse

Additional Figure S1. Western blot of podocyte proteins in urinary cell pellets from pooled urine. Protein stability for cellular pellets of selected proteins using Western analysis, with and without protease inhibitors. Cell Pellets: Lane 1, 4.8 microliters Sigma protease inhibitor; Lane 2, 4.0 microliters Sigma protease inhibitor; Lane 3, 1:100 Roche Complete tablet; Lane 4, -80˚C Frozen/Thawed; Lane 5, Fresh, no preservatives. The prototype glomerular podocyte protein of interest, podocalyxin (PODXL), was better detected from cellular pellets stored with protease inhibitors. FIBRO and smoothened (SMO) proteins were variably preserved. Sigma protease inhibitor product performed better than the Roche protease inhibitor cocktail.

Additional Figure S2. (**A**) The MLPA and long-range PCR analysis of *PKD1* DNA in urinary cell pellets**.** Long-range PCR of exons 13-15 (~5kb) and the nested PCR from a 1:1000 dilution of the long-range product. Nested PCR product for PKD1 Exon 15 part 5. (~700kb). The DNA ladder is seen in the second photograph. The loading order was Blood, Saliva, Urine, for patient R2140. **Comparison analysis of RNA**. Quality RNA was obtained from **(B)** control (RNA isolated from CHO cell line), **(C & D)** urine cellular pellets, **(E)** exosomes. RNA integrity numbers are shown from representative samples. **(F)** Impact on urine volume on reproducibility of mRNA yield for podocin, nephrin, aquaporin2, TGF β1 transcripts and **(G)** Number of samples (percent) with undetectable transcripts (patients vs. controls).

Additional Figure S1

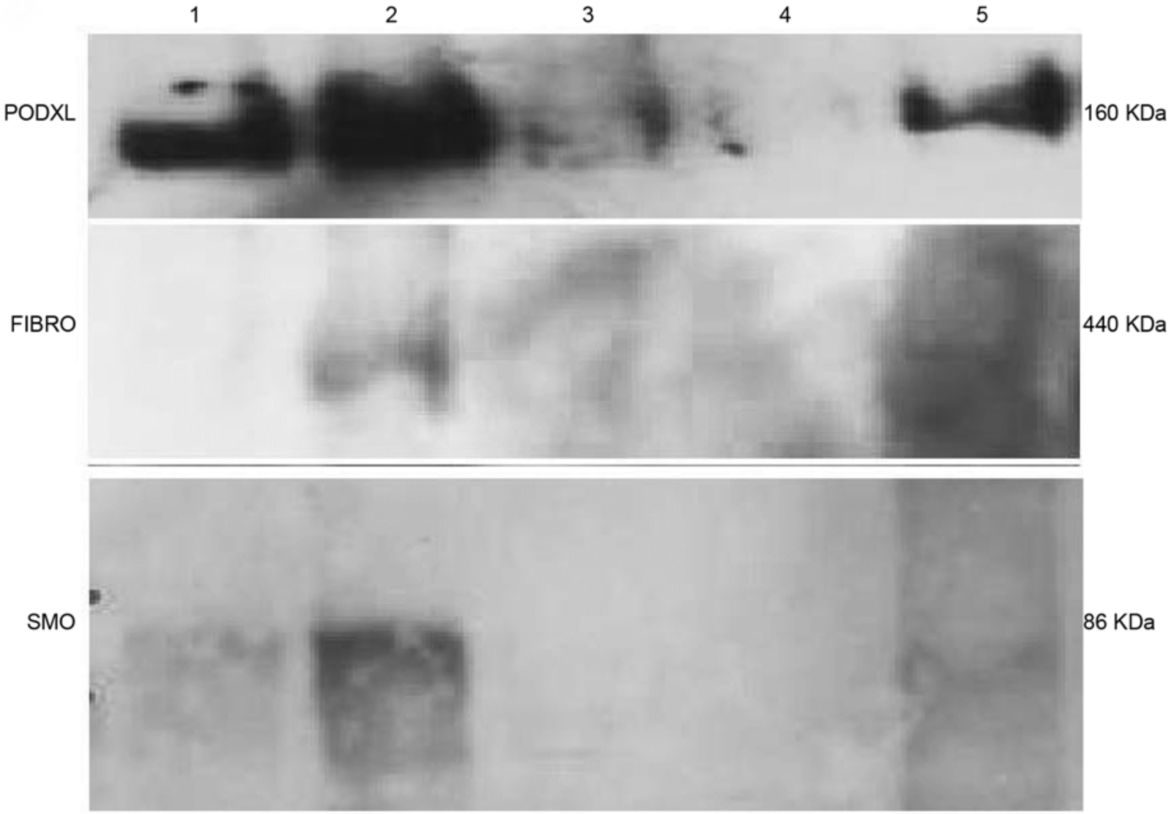

Additional Figure S2

S2A

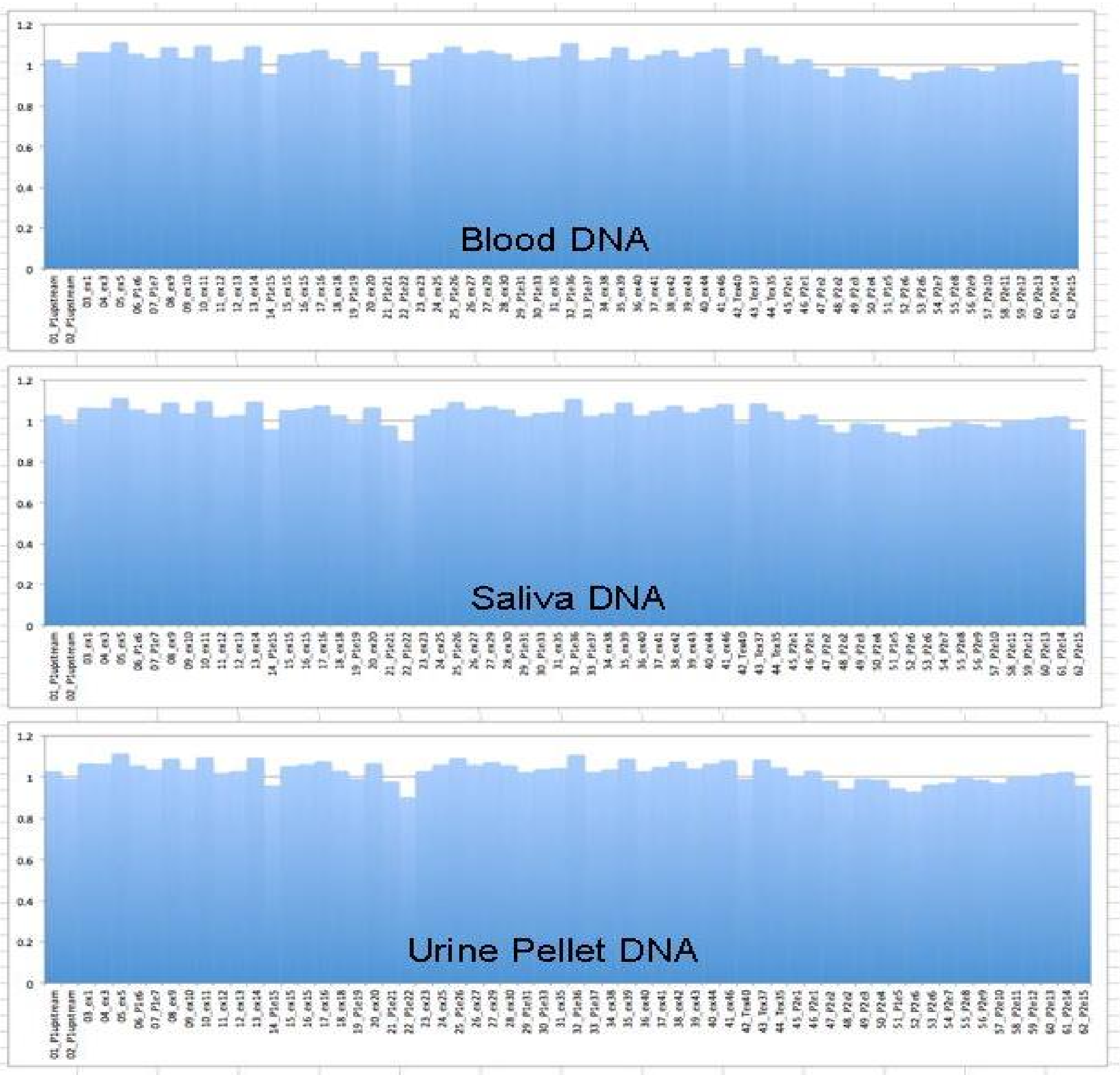

Long Range PCR
PC1 Exon 13-15

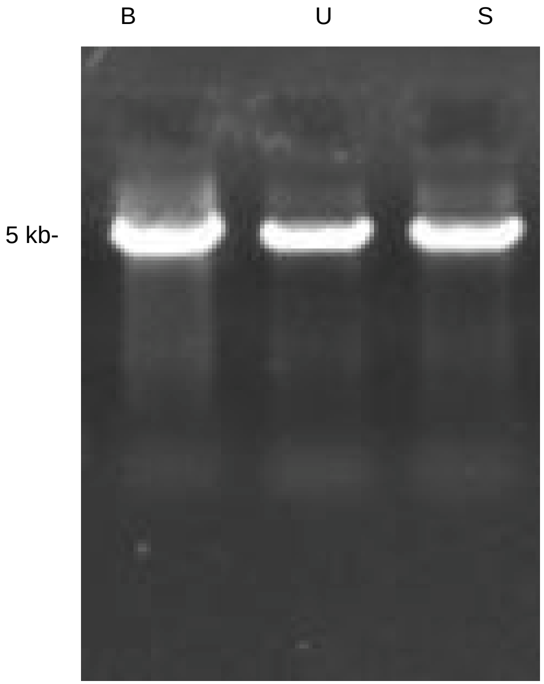

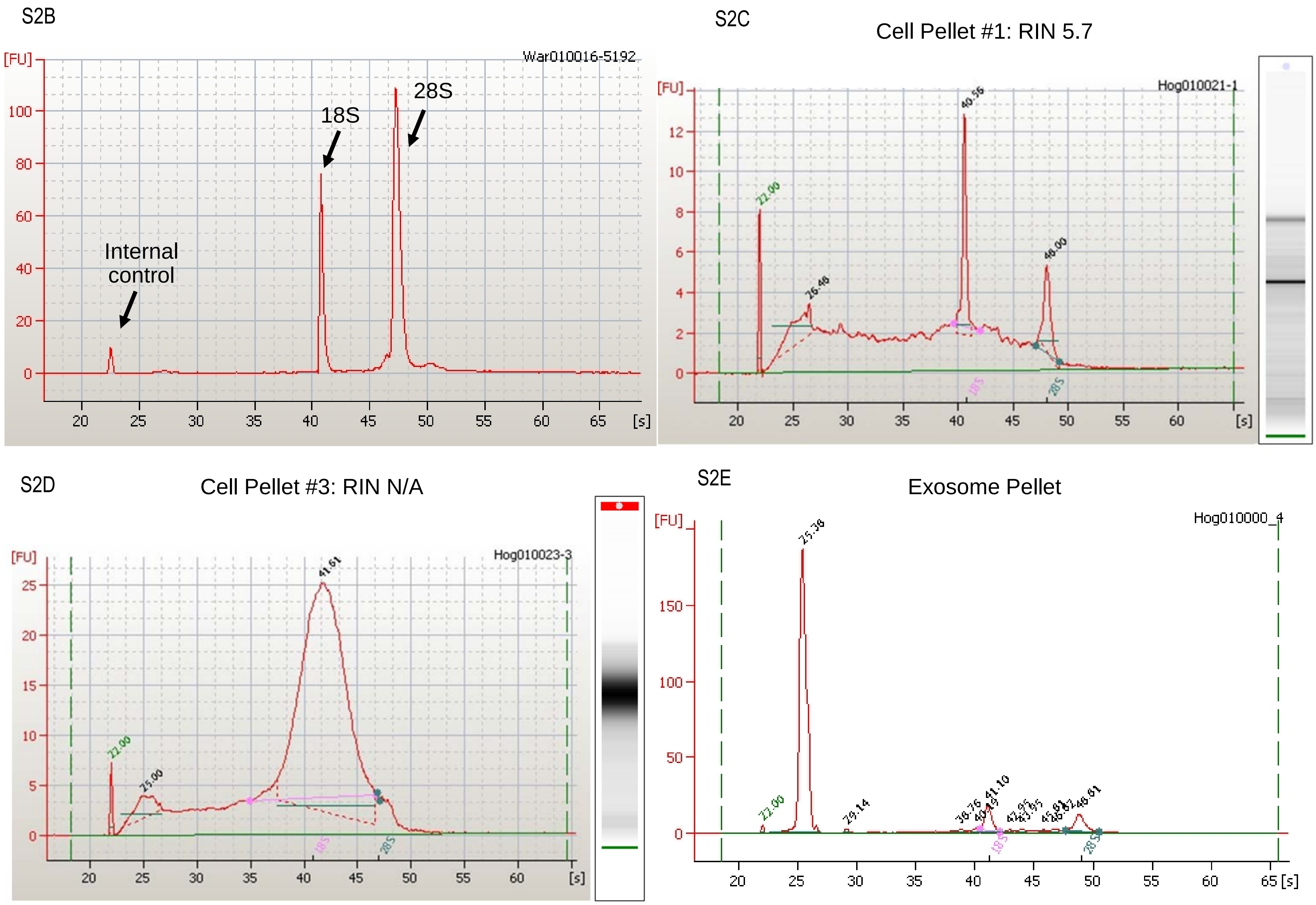
S2B
[FU]
War010016-5192
18S
28S
Internal control
[s]
S2C
Cell Pellet #1: RIN 5.7
[FU]
Hog010021-1
22.00
26.46
40.56
48.00
18S
28S
[s]
S2D
Cell Pellet #3: RIN N/A
[FU]
Hog010023-3
22.00
25.00
41.61
18S
28S
[s]
S2E
Exosome Pellet
[FU]
Hog010000_4
22.00
25.36
29.14
41.10
46.61
18S
28S
[s]

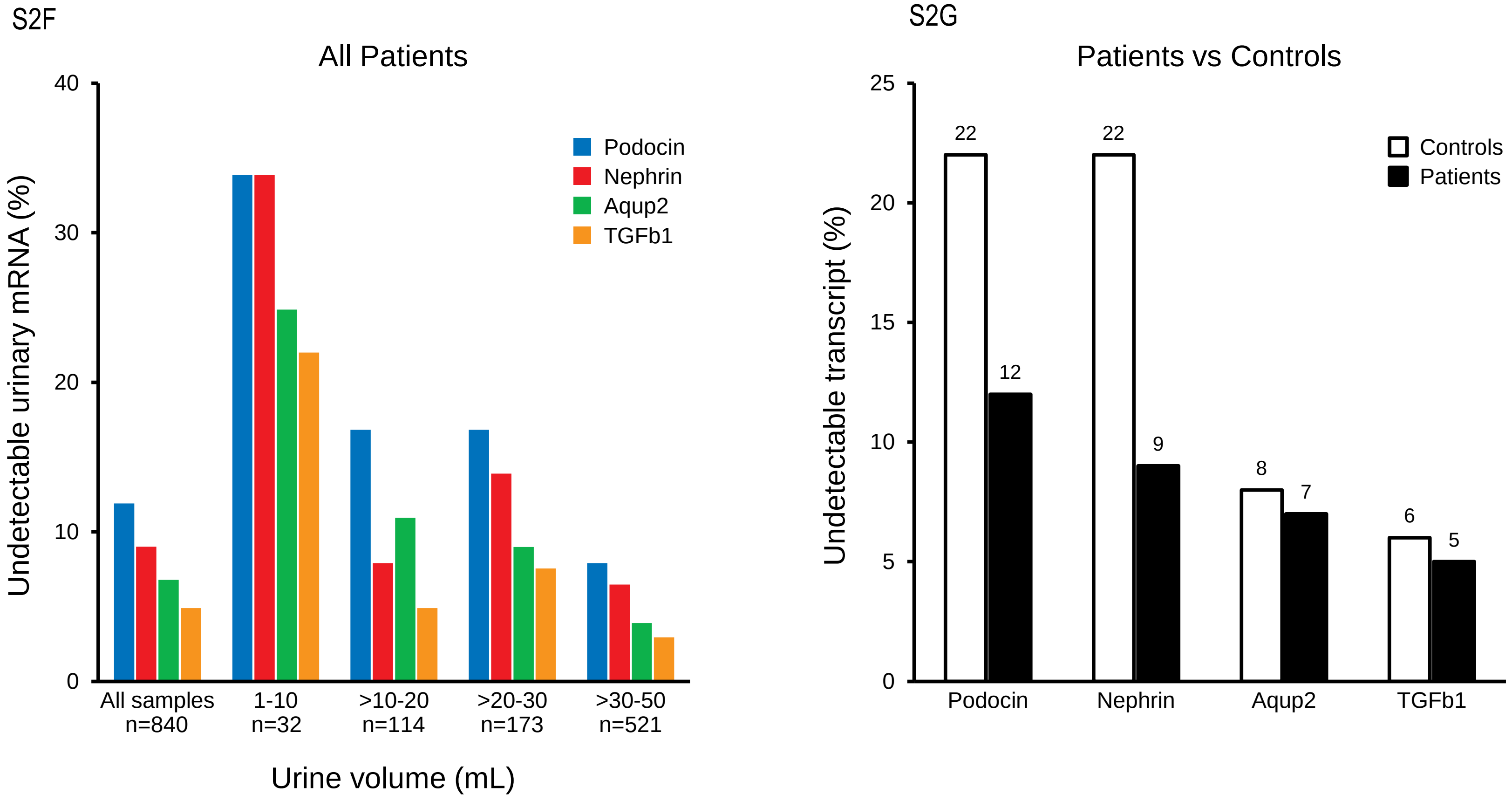
S2F
All Patients
Undetectable urinary mRNA (%)
40
30
20
10
0
Podocin
Nephrin
Aqup2
TGFb1
All samples n=840
1-10 n=32
>10-20 n=114
>20-30 n=173
>30-50 n=521
Urine volume (mL)
S2G
Patients vs Controls
Undetectable transcript (%)
25
20
15
10
5
0
Controls
Patients
22
12
22
9
8
7
6
5
Podocin
Nephrin
Aqup2
TGFb1

Additional Methods

Urine Collections

Timed urine collections are as reported in the original study protocol Version 2.5 for this study. Cell pellets were spun at 1000 g for 12 minutes. Samples are barcoded, the label providing a unique aliquot identifier number, study number assignment, participant ID number, visit number, aliquot type and aliquot number.

Procedure for spot urine processing:

Procedure for 24- hour urine sample processing:

Urinalysis

This is site specific, but typically the data collection would include the opportunity to obtain color, appearance, specific gravity, pH, leukocyte esterase, nitrite, protein, glucose, ketones, urobilin, bilirubin, and blood.

Exosome Isolation and Storage

Fresh urine (270 mL) was obtained from seven individuals and prespun at 3000 g to remove cells and debris, then ultracentrifuged at 150,000 g for 1hour to pellet a mixture of Tamm Horsfall protein (THP) and exosome like vesicles (ELVs), termed “crude exosomes.” The exosomes were frozen at -80ºC until use. For raw urine storage studies, we employed 400 mL fresh urine collected with complete protease inhibitor and 0.1% sodium azide and created 10 x 20 mL aliquots each. The visit zero urine was immediately centrifuged for 1 hr, then filtered to obtain crude exosomes; we resuspended the exosome pellets in 100uL 0.25M sucrose. The other aliqouts were stored at RT, 4ºC and -80ºC then were retrieved for exosome isolation and analysis by Western using 10 µL of exosome preparation loaded in each lane. For the long-term storage study, we took 6 samples of frozen exosomes that were frozen for >1 year after isolation from 50 mL of whole urine. After centrifugation at 4000 g for 15 min to remove cell pellet, the supernatant was centrifuged at 40,000 g for 2 hours to obtain the exosomes. The exosomes were frozen at -80ºC until needed. For the

thaw process, 50 mL frozen urine was thawed at 4ºC overnight, then vigorously vortexed and exosomes extracted per our standard ultracentrifugation protocols. The exosomal proteins PODXL, PKHD1, PKD1 were examined under similar conditions. Western analysis compared freshly extracted exosomes to those extracted from urine at RT for 24 hours or stored at 4°C x 24 hours, without protease inhibitors and sodium azide, since this preservative is used in our working protocol.

Western Blotting: Cell pellets were centrifuged at 4000 g for 20 minutes. Antibodies used in these studies were PLSCR1 (1E11) (Novus Biological, H00005359-M12), 1:5000. Secondary antibody goat anti-mouse IgG1, Southern Biotech (1070-05), 1:2000. PODXL like (3D3 monoclonal antibody), University of Michigan, 1:1000, 1 hour, wash 5 min x3; secondary goat anti mouse IgG1 (1070-05 Southern biotech at 1:2000), 1 hour, wash 5 min x3. PKHD1 antibody from Chris Ward, 1:500 dilution. Secondary antibody goat anti-mouse IgG1, Southern Biotech (1070-05), 1:2000. Smoothened monoclonal antibody from Chris Ward (generated in house) was used at 1:500 dilution. Secondary antibody Goat anti-mouse IgG2, Southern Biotech (1080-05), 1:2000. PKD-1(7e12) antibody from Chris Ward was used at 1:2000 dilution with a secondary antibody goat anti-mouse IgG_1, Southern Biotech (1070-05), 1:2000. We also used anti-podocin antibodies (Sigma; (H-130) sc-21009 (200 µg/mL). Gel: NuPage 4-12% Bis-Tris Gel, Life Technologies (Invitrogen), NP0321BOX. Gels were run at 200 volts x 55 minutes. They were then transferred to PVDF membrane: Immobilon Transfer Membrane, Millipore, IPVH00010. The transfer was run at 30V for 90 minutes.

RNA: Total RNA was isolated from cellular pellets and exosome fractions using the Qiagen RNeasy mini kit (a column extraction method) according to the manufacturers' instructions using the QIAcube automated sampler. A NanoDrop 2000C Spectophotometer (Thermo Scientific) was used for RNA analysis. Manufacturer's instructions were followed with the exception of DNase treatment.

DNA Isolation From Urine or Buccal Samples: First morning void urine samples were spun at 4,000 g x 20 minutes to collect a cell pellet, which was resuspended in 1 mL cell lysis solution, transferred to a 15 mL falcon tube, and Proteinase K (5 µL reconstituted in 50mM Tris-Hcl pH 8; 10mM $CaCl_2$) was added. Samples were incubated at 55’C overnight, then continued with standard Puregene DNA (Qiagen) isolation protocol, then cooled to RT by placing on ice for 2 min. Total volume was then divided into four 1.5 mL tubes (500 uL each). 300 uL of protein precipitation solution was added to each tube. Samples were vortexed for 30 sec, incubated on ice for 5 min, then centrifuged at 14,000 g for 1 min. (A tight white protein pellet was visible; if any additional protein floated in the tube, supernatant (SN) was transferred to a new tube and respun). Supernatant was transferred to a new 1.5 mL tube and 600 uL of 100% Isopropanol was added. Invert 30 times (Strands of DNA should be visible). Spool out DNA with a bacterial culture loop, collect DNA from all tubes and place in a new 1.5mL tube. Let air dry for 30 min. Alternatively, if DNA quantity is not sufficient to spool; centrifuge at 14,000g for 1 min to pellet DNA. Pour off 100% Isopropanol. Add 600uL of 70% EtOH and invert to wash. Centrifuge at 14,000g for 1 min, discard EtOH and drain on a paper towel for 30min or until the tube is free of EtOH. Continue to DNA rehydration step by adding 30ul DNA Rehydration Solution and let sit at 65 for 1 hour then overnight at RT (Volume added may vary depending upon amount of DNA recovered). Dilute to a final concentration of 500ng/uL and store DNA at 4’C.

Analytic Urinary NGAL Assay: NGAL was measured with rapid ELISA kits from BioPorto Diagnostics as per the manufacturer's recommendations. Standards, controls and samples were thawed, mixed, centrifuged and then plated using an XIRIL Neon 100 robotic pipetting station. Plate washing was accomplished utilizing a Thermo Wellwash AC automated plate washer. Analysis was conducted using a Molecular Devices SpectraMax 340PC microplate reader and SoftMax pro software.

MLPA: Three urine samples were used of 90 mL volume and we used the MRC Holland Kit (Cat No.P351(PKD1) and 352 kits (PKD1 and PKD2 exons) ran on ABI3730 sequencer.

Biorepository: The NEPTUNE Central Biorepository is located at University of Michigan. Urine samples for this study will also be stored at the NIDDK biorepository and at Mayo Clinic.

Procedure Class:	SPECIMENS
Procedure:	BIO-Spot Urine (SU, AS, AQ and AP)

26.B.1. UPDATES TO SPOT URINE PROCESSING PROCEDURES:

Effective 11/2012

1. Research Coordinators (RCs) will no longer fill sets of cryovials with equal volumes of sample. Effective 11/2012 please fill each cryovial with the maximum volume (1.6 mL) in sequential order (based on the aliquot number indicated on barcoded label) until all corresponding urine sample has been aliquotted. Discard remaining empty cryovials on-site.

2. When transferring urine from the original collection container or the processing tube for freezer storage, it is imperative that these containers are not overfilled. The following procedures take into account liquid expansion when samples freeze.

   a) <u>Filling cryovials with biospecimens:</u>

      - Digital pipettors: Transfer a total of 1.6 mls of sample into the cryovials.
      - Transfer pipettes (disposable, plastic): Note the two ridges below the cap of the cryovial. Use the lower ridge as a reference point for filling cryovials. Please see Figure 1 below.

When transferring urine samples from the centrifuge tubes into cryovials for freezer storage, it is imperative that these containers are not overfilled. The following directions take into account urine volume expansion when frozen.

Figure 1: Filling of Cryovials

The small, lowest ridge on the cryovial should be used for reference. Fill cryovial to this point, or with 1.6 mLs of sample.

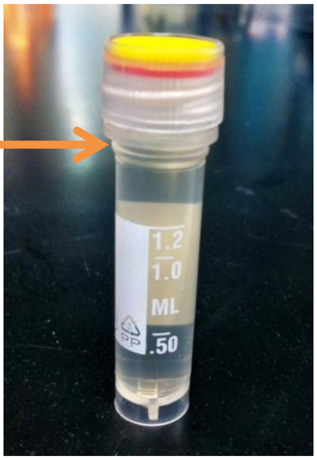

Procedure Class:	SPECIMENS
Procedure:	BIO-Spot Urine (SU, AS, AQ and AP)

26.B.2. Spot/Random Clean Catch Urine Specimen Processing Procedure

Procedure Overview
This procedure describes the process for preparing one whole urine sample type (U), two urine supernatant sample types (S, Q) and 2 urine pellet sample types(AP-E and AP-Q) from a spot (random) urine sample for NEPTUNE storage at -80° C. Shipping details are provided in Appendix N of the NEPTUNE Manual of Procedures.

Spot urine is collected in the same fashion for both pediatric and adult study participants. Spot urine samples are obtained at each study visit, including the baseline [V2] and the Biopsy [V3] visits, and all follow-up visits.

Required Supplies:
Included in kit:

- 2 x 15.0 mL centrifuge tubes[1] empty (marked **"EMPTY"**)
- 2 x 15.0 mL centrifuge tubes containing 15 μL of Protease Inhibitor[2] (marked **"PI"**)
- 16 x 1.8 mL cryovials[3] with yellow caps
(Pre-labeled as follows)

Sample 1:
SU = Whole, Unprocessed Urine

4 pre-labeled, empty cryovials
U-Spot Urine Spot Wh

SU
107658 107658
26609-Neptune
000-000
Study Visit
U-Spot Urine Spot Wh

Sample 2:
AS = Processed & NaAzide

4 pre-labeled cryovials containing Sodium Azide[4]
AS Urine Spot SN NaN3

AS
107658 107658
26609-Neptune
000-000
Study Visit
AS Urine Spot Sn NaN3

Sample 3:
AQ = Processed & Protease Inhibitor (PI)

4 pre-labeled cryovials for Protease Inhibitor
AQ Urine Spot SN PI

AQ
107658 107658
26609-Neptune
000-000
Study Visit
AQ Urine Spot Sn PI

Sample 4:
AP-E = Urine Pellet from 'Empty' centrifuge tubes

2 pre-labeled, empty cryovials
AP-E Urine Pellet

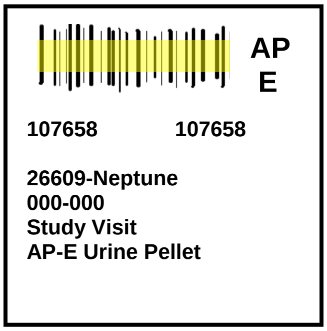

Sample 5:
AP-Q = Urine Pellet from 'PI' containing centrifuge tubes

2 pre-labeled, empty cryovials
AP-Q Urine Pellet

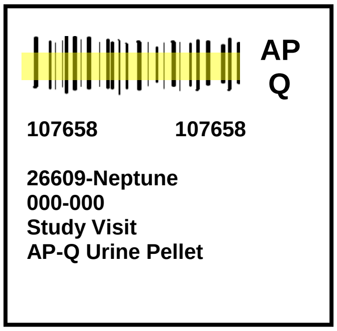

- RNA-Later[5] (expected use per sample: 50-100 μL)

Provided by site:

- Spot Urine sample from study participant
- Pipettes and tips or disposable pipettes (10 ml, 5 ml, 1 ml sizes) or plastic Transfer pipettes
- Gloves, goggles, and lab coat

Required Equipment:

- Centrifuge capable of achieving 2000 x G
- University of Michigan OSEH guidelines designate all work done with biological specimens that could produce spray from pipetting be done in a hood. Please refer to your site specific OSEH policies.

Responsible Individuals
The NEPTUNE Research Coordinator is responsible for retrieving urine sample from consented participants, returning sample to lab, and processing for storage. In the absence of the Research Coordinator, the Site PI or designated, NEPTUNE trained alternate would provide this service.

Procedure:

1. Midstream urine is recovered in sterile specimen container and stored on ice. A minimum of 32 mL of urine is necessary.
2. Note time, fasting state and 1st, 2nd, 3rd, etc urine of the day on study visit worksheet (if patient is able to provide this detail, otherwise, indicate "No recall").

Procedure Class:	SPECIMENS
Procedure:	BIO-Spot Urine (SU, AS, AQ and AP)

If processing is not able to be completed immediately, please store on ice or refrigerate for a maximum of 4 hours.

Sample processing:

Sample 1

*Requires total spot urine sample of 60 mL minimum; if sample is 50 mL or less please note in worksheet and CRF and do NOT store spot urine for the U-Spot Samples: * NO WHOLE URINE SAMPLE**

3. Using a pipettor, transfer ***whole***, unprocessed urine into the cryovials labeled:

'SU' to the right of the barcode

4. Replace the cap, firmly twisting in a clockwise direction.

Samples 2 & 3

5. Pour the remaining urine into the 4 x 15.0 mL centrifuge tubes in equal 12 milliliter portions (two tubes of each marked "EMPTY" and "Protease Inhibitor (PI)")

   E.g.:

   30 mL of spot urine → 2 – 12.0 mL centrifuge tubes for spinning (Use 1 'Empty' and 1 'PI' tube)

   50 mL of urine → 4 – 12.0 mL centrifuge tube; discard remaining urine (Use 2 of each, 'Empty' and 'PI')

NOTE: Use at least ONE EACH: "EMPTY" and "PI" centrifuge tubes

6. In the tubes labeled "PI", gently invert 8-10 times to dissolve the PI completely. Remove the cap to allow any air bubbles to escape, replace cap tightly.
7. Spin all 4 tubes at 1000 X G for 12 minutes in centrifuge

Sample 2 (AS Urine Spot): From the tubes labeled "EMPTY", transfer urine using a transfer pipettor into the 4 cryovials containing a pre-measured amount of 100 mM Sodium Azide (a biocide) labeled:

'AS' to the right of the barcode

8. When opening each cryovial, take care to place the cap directly in front of the respective tube to retain pre-measured volumes for consistent concentrations in each aliquot.
9. Pipette 1.6 mLs of urine or enough urine to fill the cryovial up to the first ridge on the cryovial (see Figure 1).
10. Replace the cap, firmly twisting in a clockwise direction. After all caps are replaced, invert tubes *at least 2 times* to distribute the sodium azide prior to freezing.

Sample 3 (AQ Urine Spot): From the tubes labeled "PI", transfer urine using a transfer pipettor into the cryovials labeled:

'AQ' to the right of the barcode

11. Pipette 1.6 mLs of urine or enough urine to fill the cryovial up to the first ridge on the cryovial (see Figure 1).

Samples 4 and 5

12. Pour off remaining supernatant from all of the 15.0 mL centrifuge tubes

Procedure Class:	SPECIMENS
Procedure:	BIO-Spot Urine (SU, AS, AQ and AP)

13. For maximal drainage, gently invert centrifuge tubes on clean paper towels for 10 seconds, take care to not disturb pellet in the centrifuge tube tip.
14. With a clean pipette tip, transfer 25.0-50.0* µL RNA-Later into each centrifuge tube, take care to not touch the inside walls of the centrifuge tube.
15. Gently stir the mixture with the pipette tip, changing tips when moving between the 'Empty' tubes and the 'PI' tubes. **Do not pipette up and down to mix as this could break any cells present in the pellet.**

    Sample 4 (AP-E Urine Pellet): From the centrifuge tubes labeled "EMPTY", pipette one urine pellet from each centrifuge tube into the two cryovials labeled:

    'AP-E' to the right of the barcode

    Sample 5 (AP-Q Urine Pellet): From the centrifuge tubes labeled "PI", pipette one urine pellet from each centrifuge tube into the two cryovials labeled:

    'AP-Q' to the right of the barcode

16. Freeze all samples at -80 C.
17. Residual urine may be disposed per OSEH guidelines enforced at participating institution.

* Amount of RNA-Later will vary depending on size of pellet. RNA-Later should be added to completely submerse the pellet.

Shipping
Samples should be shipped according to the site-specific scheduled interval for shipments.

All specimens should be shipped together **on dry ice** using the shipping instructions found in Appendix N and in the study specific container provided by NEPTUNE.

Documentation
All corresponding samples for each participant ID should be stored together. If specimen does not adequately fill pre-determined number of aliquots, please document to minimize concerns regarding lost aliquots.

Complete the appropriate Biospecimen CRF corresponding to the study visit.

Procedure Class:	SPECIMENS
Procedure:	BIO-Spot Urine (SU, AS, AQ and AP)

26.B.3. References

1. 15.0 mL orange-capped Corning centrifuge tubes: Fisher Scientific No. 05-538-53D, Corning No. 430052
2. Protease Inhibitor Cocktail: Sigma-Aldrich Catalog: P1860 – 1 mL
3. Cryovials: DOT Scientific Inc. No.: T334-6SPR
4. Sodium Azide (see attached MSDS)
5. Ambion RNA-Later Solution; P/N: Am7021
6. University of Michigan Occupations Safety and Environmental Health (OSEH) guidelines indicate washing urine and its debris down laboratory sink with adequate water.

Relevant Definitions: NaN_3: Sodium Azide

Procedure Class:	SPECIMENS
Procedure:	BIO-24-Urine (U, UQ)

26.C.1. UPDATES TO 24-HOUR URINE SAMPLE PROCESSING PROCEDURES

Effective 11/2012

1. Research Coordinators (RCs) will no longer fill sets of centrifuge tubes or cryovials with equal volumes of sample. Effective 11/2012 please fill each tube/cryovial with the maximum volume (40.0 mL and 4.5 mL, respectively) in sequential order (based on the aliquot number indicated on barcoded label) until all corresponding urine sample has been aliquotted. Discard remaining empty cryovials on-site.

2. When transferring urine from the original collection container or the processing tube for freezer storage, it is imperative that these containers are not overfilled. The following procedures take into account liquid expansion when samples freeze.

   When transferring 24-hour samples into the 50 and 5 mL storage tubes, it is imperative that the tubes are not overfilled. The following directions take into account urine volume expansion when frozen.

   a) 50 mL centrifuge tubes: Do not fill beyond 40 mLs of urine (Figure 1)
   b) 5 ml cryovials: Do not fill beyond 4.5 mls of urine (Figure 2)

(Please references the images below)

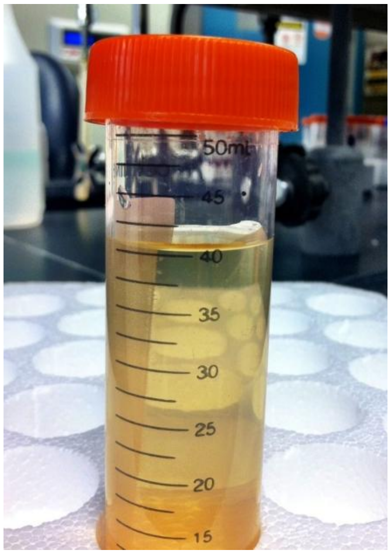

Figure 1: 50 mL centrifuge tube
Filled properly with 40 mLs of urine

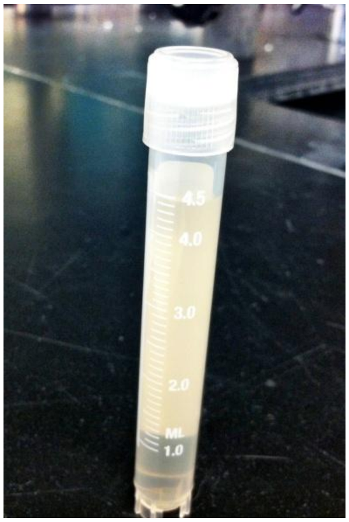

Figure 2: 5.0 mL cryovial
Filled properly with 4.5 mLs of urine

Procedure Class:	SPECIMENS
Procedure:	BIO-24-Urine (U, UQ)

26.C.2. 24-Hour or Timed Urine Processing Procedure

Procedure Overview

This procedure describes the process for preparing aliquots of the 24-hour urine sample for NEPTUNE storage at -80° C. Shipping details are provided in Appendix N of the NEPTUNE Manual of Procedures.

<u>Required Supplies:</u>

Included in kit:

- 2 x 50 mL orange top centrifuge tubes
- 5 x 5.0 mL cryovials
  (Pre-labeled as follows)
- 1 pre-labeled, empty 50 mL centrifuge tube[1] labeled as follows:

107658 107658

26609-Neptune
000-000
Study Visit
U1-24 Urine 24hr 50 ml

- 1 pre-labeled, 50 mL centrifuge tube containing 6 μL of Protease Inhibitor[2]

107658 107658

26609-Neptune
000-000
Study Visit
UQ-24 Urine 24hr 50 ml

- 5 pre-labeled 5.0 mL cryovials[3] numbered as follows:

107658 107658

26609-Neptune
000-000
Study Visit
U-24 Urine 24 Hr Wh

- Empty 24-hour urine container for tare

Provided by site:

- 24-hour urine sample from study participant
- Scale
- Pipettes and tips or disposable pipettes (10 ml, 5 ml, 1 ml sizes) or plastic transfer pipettes
- Gloves, goggles, and lab coat

<u>Required Equipment:</u>

Procedure Class:	SPECIMENS
Procedure:	BIO-24-Urine (U, UQ)

- University of Michigan OSEH[4] guidelines designate all work done with biological specimens that could produce spray from pipetting be done in a hood, please refer to your site specific OSEH policies.

Responsible Individuals
The NEPTUNE Research Coordinator is responsible for retrieving urine sample from consented participants, returning sample to lab, and processing for storage. In the absence of the Research Coordinator, the Site PI or designated, NEPTUNE trained alternate would provide this service. Local Research Coordinators need to be vigilantly aware of the participant's clinical care needs. Determine in advance if the 24-hour urine is also required for clinical care and coordinate the research sample aliquots appropriately.

Procedure

1. The participant should have been instructed on collecting a 24-hour urine sample. See Appendix K for detailed instructions on 24-hour urine collection.
2. Note start and end time for a maximum timed collection, or "first morning urine" or "random urine collection" for participants unable to provide a full 24-hour sample.

If processing is not able to be completed immediately, please store on ice or refrigerate for a maximum of 4 hours.

3. Weigh the full 24-hour urine collection and note the weight on the study visit worksheet.
4. Weigh the empty 24-hour urine collection container provided for *tare* and enter the tare value on the study visit worksheet.
5. If 24-hour urine sample is larger than one container, the samples must be combined into a single, total urine collection prior to aliquotting. (NOTE: If 2 containers are used, the tare weight must be doubled on the worksheet).

No processing should be done to the 24-hour urine sample – this should be whole, unprocessed/unspun urine.

6. Pour off two 40.0 mL aliquots into the pre-labeled centrifuge tubes:
   U1-24 Urine 24hr 50 ml and UQ-24 Urine 24hr 50 ml
7. Gently invert tube **UQ1** 8-10 times to allow dissolution of the protease inhibitor. Remove the cap to allow any air bubbles to escape, replace cap tightly. Place samples aside to complete processing the whole urine sample.
8. Pipette 5 aliquots of 4.5 mLs from the 24-hour urine sample, into the cryovials labeled:
   U-24 Urine 24hr Wh
9. Replace the caps, firmly twisting in a clockwise direction.
10. If residual urine does not need to be returned to local labs for clinical care, it may be disposed per OSEH guidelines enforced at participating institution.
11. Note time processed and freeze at -80° C.

Shipping:
Samples should be shipped according to the site-specific scheduled interval for shipments.

All corresponding specimens for each participant ID and visit should be shipped together **on dry ice** following directions in Appendix N for shipping and container provided by NEPTUNE.

Documentation
All corresponding samples for each participant ID should be stored together. If specimen does not adequately fill pre-determined number of aliquots, please document to minimize concerns regarding lost aliquots.

Complete the appropriate Biospecimen CRF corresponding to the study visit, noting any deviations from specimen collection or processing.

Procedure Class:	SPECIMENS
Procedure:	BIO-24-Urine (U, UQ)

26.C.3. References

1. 50.0 mL orange-capped Corning Centrifuge tubes: Corning no. 43290
2. Protease Inhibitor Cocktail: Sigma-Aldrich Catalog: P1860 – 1 mL
3. 5.0 mL Cryovials: Fisher Scientific No. 12-565-167N
4. University of Michigan Occupations Safety and Environmental Health (OSEH) guidelines indicate washing urine and its debris down laboratory sink with adequate water.

Relevant Definitions - None

26.C.4. For Protease Inhibitor Cocktail MSDS See 26.B.4.

24-hour Urine Sample Processing

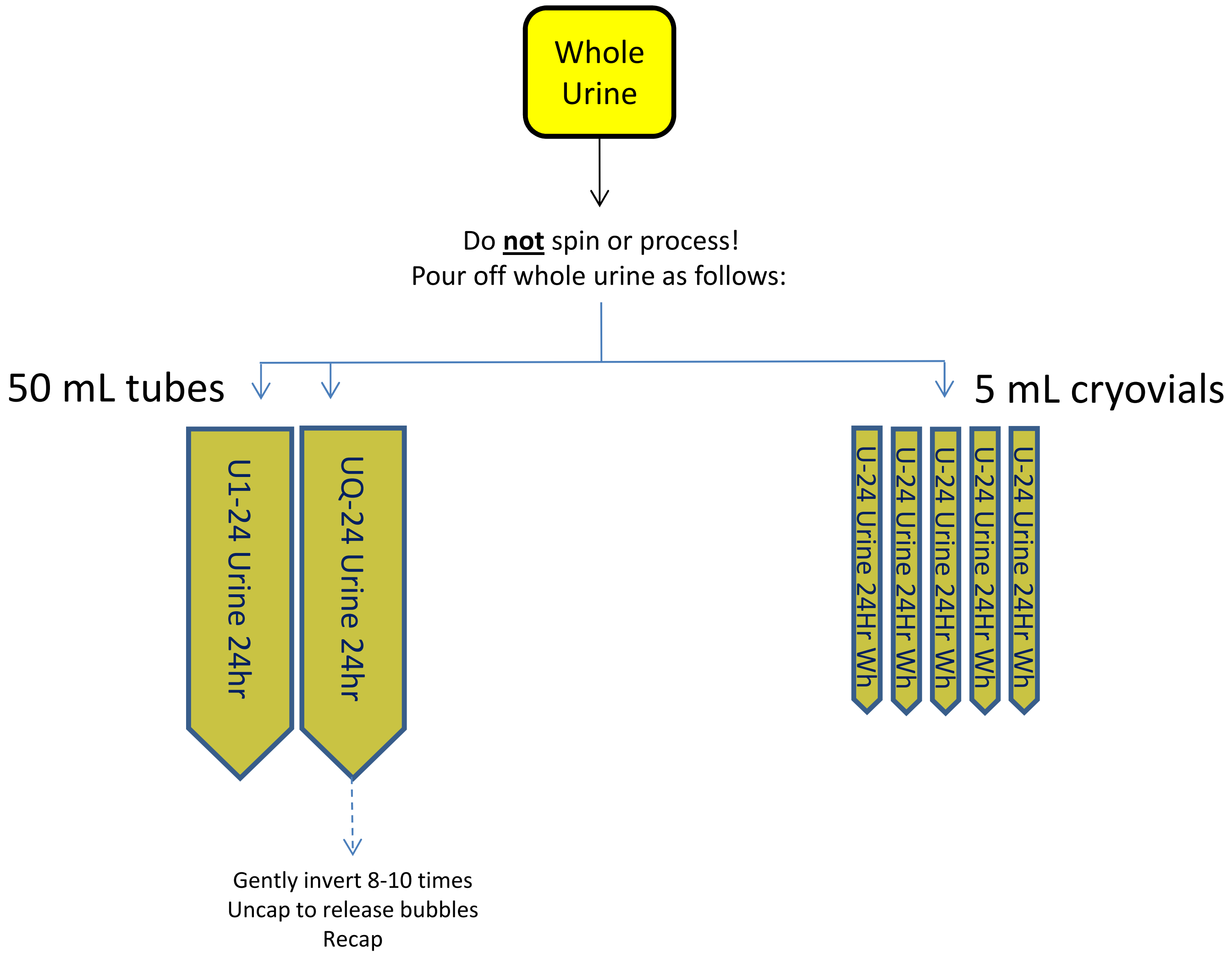

Supplement: Additional file 1: Table S1. — Standard Components of Two Commercially-Available Protease Inhibitors and Cost Analysis. Tables S2. Urine based primary outcome measures for the FSGS/MCD and MN Cohort NEPTUNE Study. Figure S1. Western blot of podocyte proteins in urinary cell pellets from pooled urine. Figure S2. (A) The MLPA and long-range PCR analysis of PKD1 DNA in urinary cell pellets. Long-range PCR of exons 13-15 (~5kb) and the nested PCR from a 1:1000 dilution of the long-range product. Nested PCR product for PKD1 Exon 15 part 5. ( ~700kb). The DNA ladder is seen in the second photograph. The loading order was Blood, Saliva, Urine, for patient R2140. Comparison analysis of RNA. Quality RNA was obtained from (B) control (RNA isolated from CHO cell line), (C & D) urine cellular pellets, (E) exosomes. RNA integrity numbers are shown from representative samples. (F) Impact on urine volume on reproducibility of mRNA yield for podocin, nephrin, aquaporin2, TGF β1 transcripts and (G) Number of samples (percent) with undetectable transcripts (patients vs. controls). Additional Methods. [file 12882_2015_185_MOESM1_ESM.pdf]
